# Electroacupuncture Regulates Inguinal White Adipose Tissue Browning by Promoting Sirtuin-1-Dependent PPAR*γ* Deacetylation and Mitochondrial Biogenesis

**DOI:** 10.3389/fendo.2020.607113

**Published:** 2021-01-21

**Authors:** Qianqian Tang, Mengjiang Lu, Bin Xu, Yaling Wang, Shengfeng Lu, Zhi Yu, Xinyue Jing, Jinhong Yuan

**Affiliations:** Key Laboratory of Acupuncture and Medicine Research of Ministry of Education, Nanjing University of Chinese Medicine, Nanjing, China

**Keywords:** electroacupuncture, obesity, WAT browning, mitochondrial biogenesis, PPAR*γ* deacetylation

## Abstract

**Background:**

Previous studies had suggested that electroacupuncture (EA) can promote white adipose tissue (WAT) browning to counter obesity. But the mechanism was still not very clear.

**Aim:**

In this study, we aim to study the effect of EA on promoting inguinal WAT (iWAT) browning and its possible mechanism.

**Method:**

Three-week-old rats were randomly divided into a normal diet (ND) group and a high-fat diet (HFD) group. After 10 weeks, the HFD rats were grouped into HFD + EA group and HFD control group. Rats in the EA group were electro-acupunctured for 4 weeks on Tianshu (ST25) acupoint under gas anesthesia with isoflurane, while the rats in HFD group were under gas anesthesia only. Body weight and cumulative food intake were monitored, and H&E staining was performed to assess adipocyte area. The effect of EA on WAT was assessed by qPCR, immunoblotting, immunoprecipitation and Co-immunoprecipitation. Mitochondria were isolated from IWAT to observe the expression of mitochondrial transcription factor A (TFAM).

**Results:**

The body weight, WAT/body weight ratio and cumulative food consumption obviously decreased (P < 0.05) in the EA group. The expressions of brown adipose tissue (BAT) markers were increased in the iWAT of EA rats. Nevertheless, the mRNA expressions of WAT genes were suppressed by 4-week EA treatment. Moreover, EA increased the protein expressions of SIRT-1, PPAR*γ*, PGC-1*α*, UCP1 and PRDM16 which trigger the molecular conversion of iWAT browning. The decrease of PPAR*γ* acetylation was also found in EA group, indicating EA could advance WAT-browning through SIRT-1 dependent PPAR*γ* deacetylation pathway. Besides, we found that EA could activate AMPK to further regulate PGC-1*α*-TFAM-UCP1 pathway to induce mitochondrial biogenesis.

**Conclusion:**

In conclusion, EA can remodel WAT to BAT through inducing SIRT-1 dependent PPAR*γ* deacetylation, and regulating PGC-1*α*-TFAM-UCP1 pathway to induce mitochondrial biogenesis. This may be one of the mechanisms by which EA affects weight loss.

## Introduction

Obesity has become an epidemic concern and brought on global public health consequences, Over one third of U.S. adults suffer from obesity (1), even 20–30% of entire populations have become obese in only 50 years (2), causing a series of severe comorbidities; for example, obesity was associated with a five-fold risk of type 2 diabetes regardless of genetic predisposition (3). Ranging from drugs to calorie restriction, the existing anti-obesity ways don’t yield the desired effect, among which white adipose tissue-browning intensified the interests in uncovering the underlying mechanisms due to its therapeutic potential for the remedy of metabolic diseases.

Adipose tissue falls into two categories: White adipose tissue (WAT) and brown adipose tissue (BAT); however the two kinds of adipose tissue have distinct functions and forms: WAT mainly stores energy as triglycerides with the feature of unicellular fat cells and single large lipid droplets, which distributes in quantity in body (4). Inversely, BAT, with non-shivering thermogenic function, is characterized by yellow-brown color on account of its plentiful content of mitochondria which are able to thermogenesis highly in virtue of the abundant uncoupling protein 1(UCP1) (5). Whereas, the thermogenic function of BAT is limited by its small proportion of total body mass (6). Indeed, it has been largely reported that WAT in rodents can undergo “browning” and acquire features of BAT following cold exposure (7), chronic endurance exercise (8, 9), and *β*3-adrenergic stimulation (10). This process had been mentioned as “browning” of WAT or WAT-browning. The interest in WAT-browning has been raised by evidence showing that WAT-browning is a new pathway in tackling metabolic diseases.

Acupuncture, as a technique of complementary and alternative therapy in the field of traditional Chinese medicine (TCM), has been applied in treating obesity for centuries in China and recognized by both the NIH and the WHO. The underlying mechanism of acupuncture for anti-obesity are researched *via* multiple pathways, including regulating lipid metabolism, modulating inflammation, suppressing appetite and promoting WAT browning (11).

Previous work has showed that EA can induce the browning of subcutaneous WAT *via* upregulating the expression of UCP1 by stimulating Zusanli (ST36) and Neiting (ST44) (12). Moreover, the study did by Lu ‘s groups implied that EA promoted the protein and mRNA expressions of UCP1, PR domain containing 16 (PRDM16), and peroxisome proliferator-activated receptor *γ* coactivator 1*α* (PGC-1*α*) in adipose tissue, and activated sympathetic nerves *via* phosphorylation of tyrosine hydroxylase (p-TH), the adenosine 2A receptor (A2AR), and *β*3-adrenoceptors (*β*3AR) in white adipose tissue (13), but the specific molecular mechanism is not clear yet, and requires more rigorous studies on it.

Mitochondria are key organelles that control the physiological role of adipocytes and normal mitochondrial function has vital effects on the health and function of adipose tissue, besides, the downregulation of mitochondrial biogenesis in obesity has long-term consequences for the metabolism of adipose tissue and the whole body (14, 15). PGC-1*α*, the co-activator of PPAR*γ*, the expression of which symbols the appearance of beige cells in white adipose tissue, participating in energy expenditure and lipid metabolism, stimulating mitochondrial biogenesis in muscle cells through an induction of the nuclear respiratory factors (NRFs) and TFAM, a direct regulator of mitochondrial DNA replication/transcription (16).

Sirtuin 1 (SIRT-1), an NAD+-dependent deacetylase, plays a vital role in regulating the browning program in WAT, which is also a principal modulator of pathways downstream of calorie restriction (17), and a key mediator of AMP-activated kinase (AMPK) action on PGC-1*α* transcriptional activity, the expression of mitochondrial and lipid metabolism genes and O2 consumption (18). SIRT-1-dependent deacetylation of PPAR*γ* on Lys268 and Lys293 makes PRDM16, a key coactivator in browning process, forming a complex with PPAR*γ*, regulating the expression of browning genes such as UCP1, then inhibiting WAT genes, and promoting the browning of WAT (19). Furthermore, the phosphorylation of AMPK upregulates its downstream regulators PGC-1*α* and SIRT-1 to increases mitochondria biogenesis (18, 20).

In this work, we aim to figure out whether EA can activate SIRT-1-dependent PPAR*γ* deacetylation pathway and PGC-1*α*-TFAM-UCP1 pathway to promote the browning of WAT and mitochondria biogenesis, so as to combat obesity.

## Materials and Methods

### Animals and Grouping

Three-week-old Sprague–Dawley male rats, suppling by the Model Animal Research Center of Nanjing Medical University and feeding in Animal Lab Center of Nanjing university of Chinese Medicine at room temperature of 28–29°C and with humidity of 40–60%, were randomly assigned to the following two groups: normal diet group (ND) and high-fat diet group (HFD). Rats in ND group were fed with normal diet, and HFD group were fed with D12451 Rodent Diet with 45 kcal% Fat (purchased from Shanghai SLAC Laboratory animal Co. Ltd). Rats were weighted each week after fasting for 8 h. After 10 weeks, obese rats were defined by a 20% increase in body weight compared to the ND ones, and then the rats in HFD group were randomly reallocated into HFD group and HFD with electroacupuncture (HFD + EA) group. The study was approved by the Institutional Animal Care and Use Committee of Nanjing University of Chinese Medicine (Animal Committee Number: 201809A015), and all procedures were conducted in accordance with the guidelines of the National Institutes of Health Animal Care and Use Committee.

### Electroacupuncture Treatment

The rats in the HFD + EA group received EA administration on ST25 (Tianshu, locating 5 mm laterally to the intersection between the upper 2/3 and the lower 1/3 in the line joining the xiphoid process and the upper border of the pubic symphysis) after gas anesthesia with isoflurane, meanwhile the same anesthesia was given to rats in the HFD groups, but without preforming EA. For the HFD + EA rats, two stainless steel acupuncture needles of 0.18 mm in diameter were inserted at a depth of 5 mm into the ST25 acupoint; the needles were connected to the output terminals of the EA instrument (Han Acuten, WQ1002F, Beijing, China), We selected continuous-wave stimulation at a frequency of 2/15 Hz (intensity 2 mA). An individual EA session was administered daily for 30 min, 6 days per week, for a total of 4 weeks. Body weight and cumulative food intake were monitored every week. After 4-week EA treatment, all the rats were sacrificed with pentobarbitone so as to collect WAT and BAT samples.

### Hematoxylin and Eosin Staining of White Adipose Tissue

Adipose tissues were taken from all the animals and fixed with 4% paraformaldehyde, which were embedded in paraffin and then sectioned at 8-μm thickness. Hematoxylin and eosin staining (H&E staining) were performed on the slides. H&E staining was performed to assess adipocyte size. The tissue slides were incubated for 5 min in the hematoxylin solution. After water ﬂushing and adding 0.5% ammonium hydroxide for 30 s, the tissue slides were put in 0.5% eosin for 2-min dyeing and measured under microscope (Nikon TE2000, Japan). We used Image-Pro Plus software to collect image fields of each rat for adipocyte area, and more than 250 cells per rat were selected for measuring (13).

### Immunoblotting

The total protein of inguinal white adipose tissue was extracted using the Adipose Protein Extraction Kit (Minute AT-022). BCA Protein Assay Kit (23227) was used to measured protein concentrations. Then, 20 µg of protein was separated by SDS-PAGE and transferred to PVDF membranes. After blocking for 2 h with 5% BSA, the membrane was then probed with primary antibodies under 4°C overnight. Reaction with the corresponding secondary antibodies was performed at room temperature for 1 h. Sources of antibodies are shown in **Table 1**.

**Table 1:** Antibodies.

Antibodies	Source	Item No.
**UCP1**	Abcam	ab10983
**SIRT-1**	CST	9475s
**PRDM-16**	Abcam	ab106410
**AMPK**	CST	2532s
**P-AMPK**	CST	2535s
**PGC-1α**	Abcam	ab54481
**PPARγ**	Santa-Cruz	SC-7273
**TFAM**	Abcam	ab131607
**Acetylated-Lysine**	CST	9441s

### RT-PCR

Total RNA was extracted from 200 mg of IWAT using Trizol (TaKaRa, Japan). The total RNA was reverse-transcribed to produce cDNA by a thermal cycler (Bio-Rad, United States) with Prime Script RT Master Mix (TaKaRa, Japan) at 37°C for 15 min and 98°C for 15s. The PCR reaction mixture (20 µl) contained of 4 µl of forward primer, 4 µl of reverse primer, 2 µl of cDNA, and 10 µl of SYBR Green Mix (TaKaRa, Japan).The PCR protocol was as follows: 40 cycles of ampliﬁcation for 30 s at 95°C, 5s at 95°C, 30 s at 60°C. The primers used in the experiment are shown in **Table 2**.

**Table 2:** Primers used in this study.

Genes	Forward	Reverse
**Cox8b**	CAGTGATTCCTAAAGCCCGTAT	GATTATGACTGACATCCCCACA
**TMEM26**	ACCTGGGTGAAGGAAGAGCA	GGAGAAAGCCATTTGTAGCC
**DIO2**	ACAGGTTAAATTGGGTGAAGAT	GCAAAGTCAAGAAGGTGGC
**Metrnl**	CAAGGAGGTGGAGCAGGTGTA	CTGGGCAGGTGAGAAGGTGTT
**Slc25a44**	GCAGGTTGAAGGCAAGAGC	TGGAGGGTGTAGCCGAGAT
**EPDR1**	GGGTGCTGGACGAAAGGA	CAGGGTTCTGCCAAGGGTA
**TBX1**	TATGGGACGAGTTCAATCAGC	TGTCATCCACAGGCACAAAGT
**TBX15**	ACGTGATCCGCAAGGACTTC	TGATAGGCTGTGACTGTGGTAAA
**HOXC9**	GCTACATGCGGACTTGGC	TCTCCTCTTTGTGCTTGCTG
**CD137**	TTTTACAACCCTGCTCCGT	AGAAGCCTTGCTCCTCTACC
**Cidea**	TCATACACCCTGCTCGTCCT	CCAGAGTCTTGCTGATAAGTTCC
**Elovl6**	ACATCTTCTGGTCCTCACTCAT	CCTTCGTCGCTTTCTTCACT
**Resistin**	GGACGGTTGATTGAGAACTGAGC	ACCACCATCATCCCATTGTGTATT
**Agt**	CGCGTATACATCCACCCCTTTCA	CGATCCTCAGCCTCTAGCTTCTCA
**Ednra**	AGTGGAAGAACCAGGAGCAGAAC	GACAAAAAGCAGGGGAGAGACC
**Timp2**	GACACGCTTAGCATCACCCAGA	CCATCCAGAGGCACTCATCCG
**Itga6**	CTCAGTATTCAGGGATAGCGTG	TGTGGTAGGTGGCATCGTAA

### Isolation Mitochondria From Inguinal Adipose Tissue

Differential centrifugation was used to isolate mitochondrial from Inguinal adipose tissues. After rinsing and weighing, tissues were homogenized and suspended in pre-chilled isolation buffer (1 ml/100 mg tissue) contained with 250 mM sucrose, 5 mM HEPES, 2 mM EGTA, 2% fatty acid-free BSA, PH 7.2. The homogenized tissue was centrifuged at 900 ×g for 10 min at 4°C, then the supernatant was transferred into a new pre-cold 10 ml tube, leaving the fat layer in the original tube and re-suspending with isolation buffer. The two tubes were centrifuged again at 900× g for 10 min at 4°C and then transferred into 2 ml tubes and centrifuged twice at 9,000 ×g for 10 min at 4°C. After that, the pellets were re-suspended in SHS without BSA, and protein concentration was determined by BCA (21).

### IP and Co-IP

Protein was precleared using protein A/G agarose beads and incubated with anti-acetylated lysine antibody or PPAR*γ* antibody overnight at 4°C. then protein A/G agarose beads were added and incubated for 2 h at 4°C. Beads were pelleted by centrifugation and washed three times with lysis buffer and resolved on SDS-PAGE and analyzed by immunoblotting with PPAR*γ*, PRDM-16 or PGC-1*α* antibodies.

### Statistical Analysis

The data are presented as the mean ± standard deviation (SD), performing by Prism 8.0.2, Groups were compared using one-way analysis of variance (ANOVA) followed by Dunnett t3 *post hoc* test. P <0.05 was considered statistically significant between the comparing groups.

## Results

### Electroacupuncture Treatment Significantly Reduced the Body Weight, Cumulative Food Intake, Area of Adipocytes, and the Ratio of the Weights of White Adipose Tissue to Body Weight

**Figure 1:**
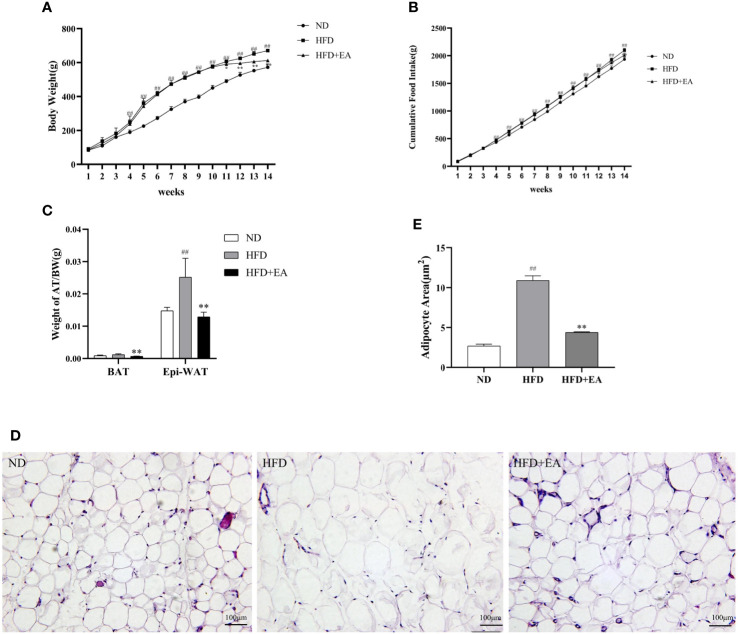
Effect of EA on body weight, food intake, adipose weight or adipocyte area. **(A, B)** Body weight and cumulative food intake of normal diet (ND), high-fat diet (HFD) and high-fat diet + EA (HFD + EA) groups during 14 weeks (n = 5). **(C)** Epididymal white adipose tissue/body weight, brown adipose tissue/body weight in the ND group, HFD group and HFD + EA group (n = 5). **(D, E)** H&;E staining and adipocyte area of IWAT adipocyte size (20×, scale bar = 100 µm) in ND, HFD and HFD + EA group. Data represent the mean ± SD (n = 3). ^##^P < 0.01 vs. the ND group; *P < 0.01, **P < 0.05 vs. the HFD + EA group.

Compared with the ND group, there was an expected enhancement in the body weight (BW) of the HFD group after 10-week high fat diet (**Figure 1A**) and EA effectively suppressed HFD-induced weight gain (**Figure 1A**). To evaluate the specific effect of EA on adipose tissue, we isolated the epididymal white adipose tissue (EWAT) and brown adipose tissue (BAT) from the rats in each group and calculated the ratios of EWAT and BAT to their individual body weight. We found that the ratio of EWAT/BW in the HFD group was higher than that in the ND group, but was significantly decreased by EA and the ratio of BAT/BW in HFD group was also reduced by EA (**Figure 1C**). After treating by EA, we observed there was a distinct decrease of the cumulative food intake in HFD+EA group compared to HFD group (**Figure 1B**), which suggested that EA may inhibit appetite to a certain extent in these rats. We analyzed the form of adipocytes by H&E staining and observed distinctive smaller area of adipocytes of the IWAT in the HFD+EA rats compared with that in the HFD rats (**Figures 1D, E**), suggesting that EA might promote lipolysis and decreasing adipocyte size in obese rats.

### Electroacupuncture Can Improve Mitochondrial Biogenesis in Inguinal White Adipose Tissue

**Figure 2:**
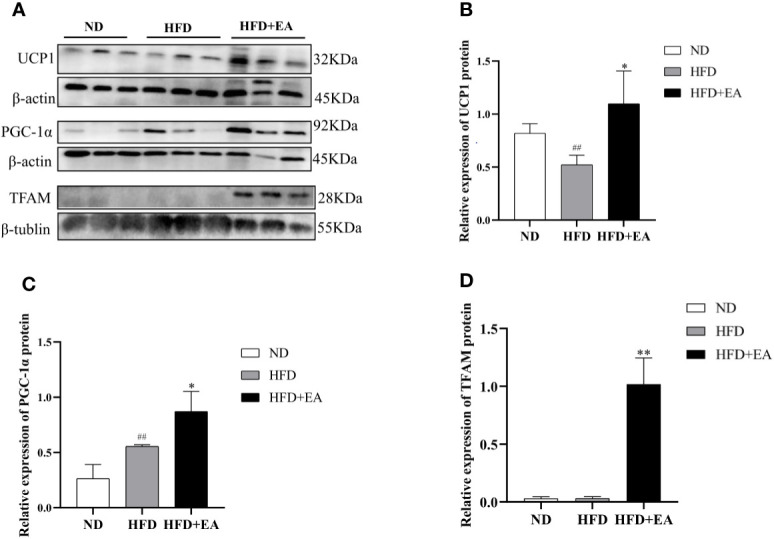
Effect of EA treatment on mitochondrial biogenesis in IWAT from ND, HFD or HFD+EA group. Western blot image of UCP1, PGC-1a and TFAM **(A)** staining and relative protein levels of UCP1 **(B)**, PGC-1a **(C)** and TFAM **(D)**. Data was represented as mean ± SD (n = 3). ^##^P < 0.01 vs. the ND group; *P < 0.01, **P < 0.05 vs. the HFD + EA group.

We can differentiate WAT from BAT by the number of mitochondrion, which is one of the most distinct features of BAT and a marker of thermogenesis. We isolated mitochondria from IWAT to observe the protein expression of TFAM using western blot, and the result showed that a 4-week EA treatment evidently increased the protein expression of TFAM, which displayed a low expression in ND and HFD groups (**Figures 2A, D**). In addition, PGC-1*α*, the main regulator during WAT mitochondrial biogenesis and BAT thermogenesis, was also significantly enhanced after treating by EA (**Figures 2A, C**). UCP1, which uncouples the electron transport chain from ATP synthesis to provide non-shivering thermogenesis in the inner mitochondrial membrane, was increased by EA obviously (**Figures 2A, B**).

### The mRNA Expressions of White Adipose Tissue Genes Were Suppressed by 4-Week Electroacupuncture Treatment, Moreover, Electroacupuncture Increased the mRNA Expressions of Brown Adipose Tissue-Specific Thermogenic Genes in Inguinal White Adipose Tissue

**Figure 3:**
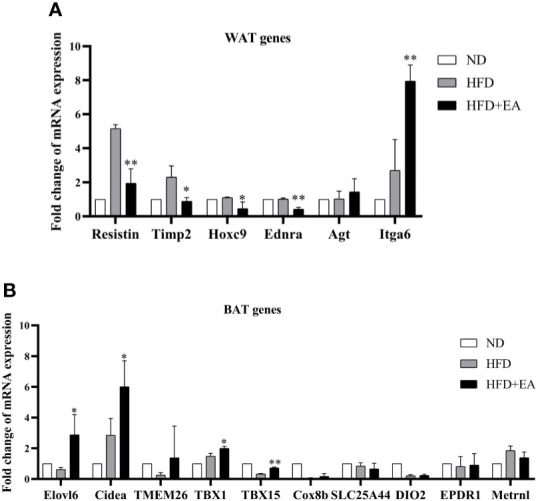
EA treatment induced the mRNA expression of WAT and BAT related genes in IWAT. Fold change of WAT **(A)** and BAT **(B)** relating genes mRNA expressions. Data were represented as mean ± SD (n = 3). ^*^P < 0.05, ^**^P < 0.01 *vs.* the HFD+ EA group.

In this study, we observed that EA made a distinct decrease in the mRNA levels of resistin, TIMP metallopeptidase inhibitor 2 (Timp2), homeobox C9 (Hoxc9) and endothelin receptor type A (Ednra), which were WAT-related genes in IWAT (**Figure 3A**). Conversely, there was a promotion in the expression of integrin subunit alpha 6 (Itga6) in the HFD + EA group (**Figure 3A**). The phenomenon of WAT-browning is associated with an increase in the expression of the BAT-specific thermogenic genes in WAT. The 4-week EA treatment obviously upregulated the mRNA levels of beige fat marker genes in IWAT, including Elov1 fatty acid elongase 6 (Elovl6), cell death-inducing DFFA-like effector a (Cidea), T-box transcription factor 1 (Tbx1) and T-box transcription factor 15 (TBX15) (**Figure 3B**).

### Electroacupuncture Increased the Expression of Proteins That Trigger the Molecular Conversion of White Adipose Tissue Browning

**Figure 4:**
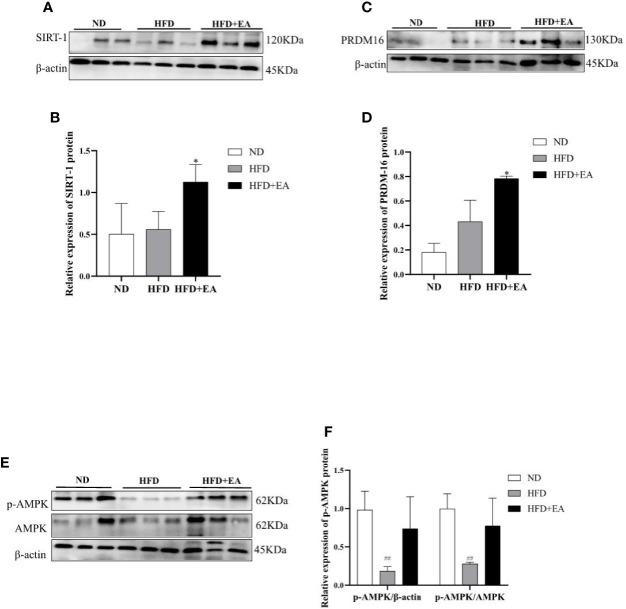
EA increased WAT-browning related proteins in IWAT of HFD + EA group but not in HFD group. SIRT-1 **(A, B)**, PRDM16 **(C, D)**, and p-AMPK **(E, F)** expression in IWAT obtained from ND-fed, HFD-fed or HFD + EA-fed rats following 14 weeks of treatment. Data were represented as mean ± SD (n = 3). ^##^P < 0.01 vs. the ND group, *P < 0.05 vs. the HFD + EA group.

We performed western blot to observe the expression of WAT-browning related protein. The protein expression of SIRT-1 in the HFD + EA group showed an apparent increase after 4-week EA treatment (**Figures 4A, B**). In addition, the key protein PRDM16 responsible for WAT-browning was notably improved in HFD+EA group (**Figures 4C, D**). Additionally, the expression of p-AMPK in IWAT was raised by EA, which was apparently decreased in HFD group (**Figures 4E, F**).

### Electroacupuncture Induced Sirtuin-1-Dependent Deacetylation of PPAR*γ* and Peroxisome Proliferator-Activated Receptor γ Coactivator-1α and Promotes PPAR*γ*-PR PR Domain Containing16 Interaction

**Figure 5:**
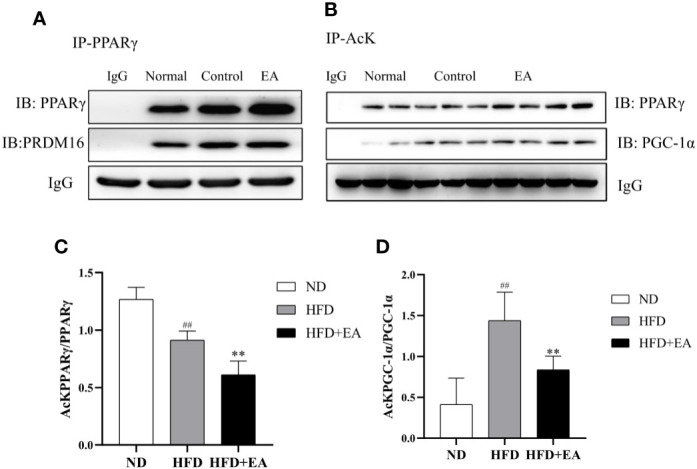
EA treatment facilitated the interaction between PPAR*γ* and PRDM-16 and decreased PPAR*γ* and PGC-1*α* acetylation. IWAT tissues from ND, HFD and HFD + EA groups were immunoprecipitated with acetylated lysine (AcK) antibody and immunoblotted with PPAR*γ* and PGC-1*α* antibodies. **(A)** Western blot for Co-IP studies of PPAR*γ* and PRDM16. **(B)** Acetylation of PPAR*γ* and PGC-1*α* in white adipose tissue. The ratios of acetylated PPAR*γ* to PPAR*γ* **(C)** and acetylated PGC-1*α* to PGC-1*α* **(D)**. Data were represented as mean ± SD (n = 3). ^##^P < 0.01 vs. the ND group; **P < 0.01 vs. the HFD + EA group.

We evaluated the acetylation status of PPAR*γ* and PGC-1*α* in each group and also determined PPAR*γ*-PRDM16 interaction by co-immunoprecipitation (Co-IP) technique. As shown in **Figure 5A**, the interaction between PPAR*γ* and PRDM16 was improved by EA treatment. We used acetylated lysine antibody to assess the acetylated states of PPAR*γ* and PGC-1*α* (**Figure 5B**); the result illustrated that EA notably reduced the ratios of acetylated PPAR*γ* and PGC-1*α* ((**Figures 5C, D**) which were elevated in HFD group.

## Discussion

Obesity poses a severe threat to worldwide public health, and the people suffering from obesity is growing in quantity at an alarming rate resulting from the convenient calorie-dense food and sedentary lifestyle. Identiﬁcation of specific molecular mechanism that EA suppresses diet-induced obesity will advance the development of effective strategies to counter obesity. The main focus of the EA anti-obesity is mostly on the crosstalk between brain and nerves, but little on the target organ-adipose tissue. In this study, we put emphasis on the WAT and the specific molecular mechanism about how the WAT-browning occurs under the treatment of EA.

The studies from clinical and experiments indicate that EA can promote peptide levels of *α*-MSH in the arcuate nuclear of hypothalamus (ARH) neurons (22), and the expression of the cocaine and amphetamine-regulated transcript (CART) peptide to approach normal level, resulting in an inhibition of food intake and a reduction of body weight in DIO rats (23), and EA can suppress appetite through regulating appetite regulatory hormones and downstream signaling pathway (11, 24). In addition, it has been reported that EA reduces body weight and food intake by up-regulating the WAT-browning related proteins UCP1, PRDM16 and PGC-1*α* of WAT in high-fat-diet induced mice (13). In this study, there were significantly reduced levels of cumulative food intake and weight gain in the HFD + EA group (**Figures 1A, B**). Moreover, the ratio of the WAT to body weight and the adipocytes area significantly decreased after EA (**Figures 1C–E**). These indicated that EA can suppress appetite, promote WAT lipolysis and restrict adipose tissue synthesis to achieve weight-loss.

It has been reported that the raise of SIRT-1-mediated PPAR*γ* deacetylation further facilitated the interaction of PPAR*γ* and PRDM16, which eventually promoted brown features in white adipocytes to prevent obesity (19). We observed the mRNA expression of BAT/Beige related-genes and WAT related-genes in IWAT by RT-PCR, the result revealed that EA treatment selectively induced the mRNA expression of BAT genes and repressed WAT genes in IWAT (**Figures 3A, B**). Furthermore, the increased protein expression of SIRT-1 was found in the HFD + EA group (**Figures 4A, B**). Consequently, we further detected the acetylated status of PPAR*γ* using acetylated lysine antibody. It demonstrated that the acetylation of PPAR*γ* was prominently decreased by EA (**Figures 5B, C**), but the binding of PPAR*γ* and PRDM16 was increased (**Figure 5A**). Besides, the protein expression of PRDM16 was effectively boosted by EA (**Figures 4C, D**). The results above indicated BAT program occurred in the HFD + EA group. For the first time, we found that EA activated SIRT-1-dependent deacetylation of PPAR*γ* to induce WAT-browning. For further study, we want to use SIRT-1 knockout mice to verify the important role of pathway.

UCP1, in the inner mitochondrial membrane, uncouples the electron transport chain from ATP synthesis to provide non-shivering thermogenesis (25). UCP1 null mice are cold sensitive and unable to maintain their body temperature (26). Mitochondria, essential organelles for maintaining the function of adipocytes in metabolic homeostasis (15), provide the largest part of cellular thermogenesis, participating in the differentiation and maturation of adipocytes (27). Mitochondrial biogenesis is a vital link in the process of WAT-browning, which is regulated by a chain of molecular mechanisms. PGC-1*α* induces its downstream activator nuclear respiratory factors 1 and 2 (NRF1, NRF2), which interactives the nucleus with mitochondrion and combine with the antioxidant response element (28), subsequently activates the TFAM (29), one of the major regulators of mitochondrial biogenesis, to trigger mitochondrial DNA replication and the transcription of mitochondrial DNA (16, 30). The activation of this PGC-1*α*-NRF-TFAM pathway leads to synthesis of mitochondrial DNA and proteins and generation of new mitochondria (31). Moreover, in adipocyte-specific TFAM-knockout mice, the activity of the proteins in complexes I, III, and IV was significantly decreased, which resulted in adipocyte death and inflammation in the WAT (32). And in high-fat-diet-fed rats, mitochondrial biogenesis and the copy number of mitochondrial DNA (mtDNA) were decreased in white adipose tissues (33). In previous researches, EA can prevent obesity to induce the expression of PGC-1*α* both in WAT and BAT (13, 34), and UCP1 expression is also remarkably elevated by EA (35, 36). We formulated a hypothesis that the PGC-1*α*-TFAM-UCP1 pathway may contribute to the induction of WAT-browning by EA treatment. The protein expression of TFAM was elevated in mitochondria of the HFD + EA group (**Figures 2A, D**). Meanwhile, EA treatment enhanced the protein expressions of PGC-1*α* and UCP1 (**Figures 2A–C**). These demonstrated that the coordination between PGC-1*α* and TFAM in the process of mitochondria biogenesis. Above all, these observations suggested that EA activated the PGC-1*α*-TFAM pathway to induce UCP1 transcription to promote mitochondrial biogenesis and adaptive thermogenesis.

AMPK, the cellular energy sensor, plays a major role in regulating cellular energy balance and is activated by low ATP (37). AMPK can activate SIRT-1 to trigger deacetylation of PGC-1*α* (18). In addition, AMPK activation also increases mitochondrial biogenesis by upregulating PGC-1*α* (20). Our study demonstrated that EA treatment could trigger phosphorylation of AMPK and then down-regulate acetylation of PGC-1*α*, suggesting the up-regulation deacetylation of PGC-1*α* by EA (**Figures 5B, D**). PGC-1*α* as a transcriptional co-activator interacts with other transcription factors, which regulate the expression of fatty acid oxidation, oxidative phosphorylation, and mitochondrial biogenesis related genes. We found beige fat marker genes, including Elov1, Elovl6, Cidea, Tbx1, and Tbx15, which may result from the activity of PGC-1*α*.

## Conclusion

**Figure 6:**
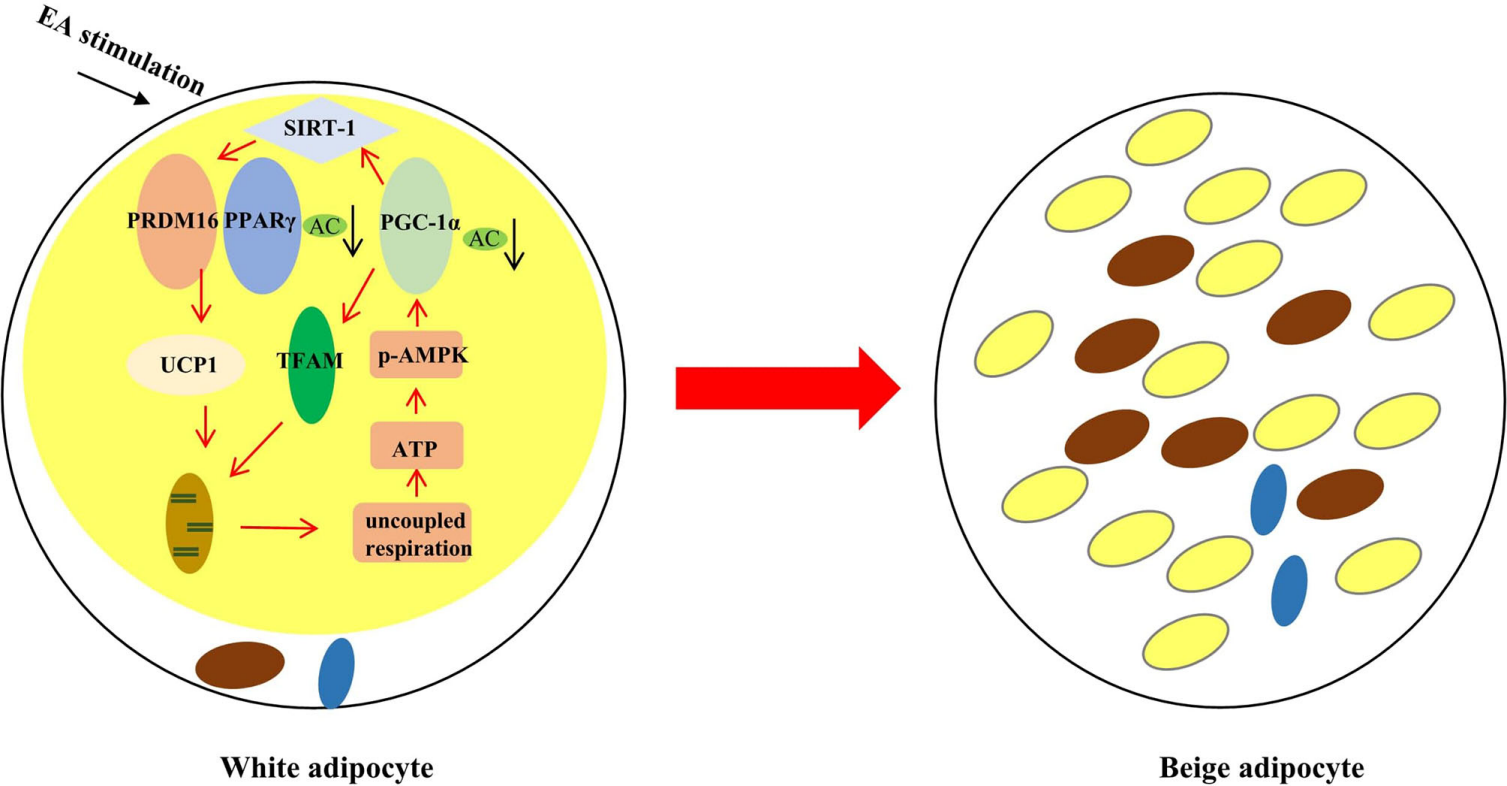
The effect of EA stimulation on WAT. EA can remodel WAT to BAT through inducing SIRT-1 dependent PPAR*γ* deacetylation, and regulating PGC-1*α*-TFAM-UCP1 pathway to induce mitochondrial biogenesis. The red rows mean promotion, and the black rows mean reduction.

Taking together (**Figure 6**), EA may regulate SIRT-1 dependent PPAR*γ* deacetylation and further improve the interaction of PPAR*γ* with PRDM16 to acquire the ability for uncoupled respiration. In addition, EA treatment can trigger phosphorylation of AMPK and then activate PGC-1*α*-TFAM-UCP1 pathway to promote mitochondrial biogenesis. These results have implications for EA treatment for obesity.

## Data Availability Statement

The original contributions presented in the study are included in the article/supplementary material, further inquiries can be directed to the corresponding authors.

## Ethics Statement

The animal study was reviewed and approved by Experimental Animal Ethics Committee of Nanjing University of Chinese Medicine.

## Author Contributions

XJ, JY, and BX conceived and designed the experiments. QT, ML, YW, SL performed the experiments, QT wrote the manuscript and XJ corrected and revised the manuscript. All authors contributed to the article and approved the submitted version.

## Funding

This work was financially supported by the National Natural Science Foundation of China (No. 81873238) and the Open Projects of the Discipline of Chinese Medicine of Nanjing University of Chinese Medicine Supported by the Subject of Academic priority discipline of Jiangsu Higher Education Institutions (No. ZYX03KF015).

## Conflict of Interest

The authors declare that the research was conducted in the absence of any commercial or financial relationships that could be construed as a potential conflict of interest.

## References

[1] Fruh SM Obesity: Risk factors, complications, and strategies for sustainable long-term weight management. J Am Assoc Nurse Pract (2017) 29(S1):S3–14. 10.1002/2327-6924.12510 29024553 PMC6088226

[2] Lean M Astrup A Roberts SB Making progress on the global crisis of obesity and weight management. BMJ (2018) 361:k2538. 10.1136/bmj.k2538 29898885 PMC5997033

[3] Schnurr TM Jakupovic H Carrasquilla GD Angquist L Grarup N Sorensen T Obesity, unfavourable lifestyle and genetic risk of type 2 diabetes: a case-cohort study. Diabetologia (2020) 63(7):1324–32. 10.1007/s00125-020-05140-5 32291466

[4] Wu J Bostrom P Sparks LM Ye L Choi JH Giang AH Beige adipocytes are a distinct type of thermogenic fat cell in mouse and human. Cell (2012) 150(2):366–76. 10.1016/j.cell.2012.05.016 PMC3402601 22796012

[5] Nedergaard J Golozoubova V Matthias A Asadi A Jacobsson A Cannon B UCP1: the only protein able to mediate adaptive non-shivering thermogenesis and metabolic inefficiency. Biochim Biophys Acta (2001) 1504(1):82–106. 10.1016/s0005-2728(00)00247-4 11239487

[6] Sepa-Kishi DM Ceddia RB White and beige adipocytes: are they metabolically distinct? Horm Mol Biol Clin Invest (2018) 33(2). 10.1515/hmbci-2018-0003 29466235

[7] Barbatelli G Murano I Madsen L Hao Q Jimenez M Kristiansen K The emergence of cold-induced brown adipocytes in mouse white fat depots is determined predominantly by white to brown adipocyte transdifferentiation. Am J Physiol Endocrinol Metab (2010) 298(6):E1244–53. 10.1152/ajpendo.00600.2009 20354155

[8] Wu MV Bikopoulos G Hung S Ceddia RB Thermogenic capacity is antagonistically regulated in classical brown and white subcutaneous fat depots by high fat diet and endurance training in rats: impact on whole-body energy expenditure. J Biol Chem (2014) 289(49):34129–40. 10.1074/jbc.M114.591008 PMC4256346 25344623

[9] De Matteis R Lucertini F Guescini M Polidori E Zeppa S Stocchi V Exercise as a new physiological stimulus for brown adipose tissue activity. Nutr Metab Cardiovasc Dis (2013) 23(6):582–90. 10.1016/j.numecd.2012.01.013 22633794

[10] Park JW Jung KH Lee JH Quach CH Moon SH Cho YS 18F-FDG PET/CT monitoring of beta3 agonist-stimulated brown adipocyte recruitment in white adipose tissue. J Nucl Med (2015) 56(1):153–8. 10.2967/jnumed.114.147603 25525187

[11] Wang LH Huang W Wei D Ding DG Liu YR Wang JJ Mechanisms of Acupuncture Therapy for Simple Obesity: An Evidence-Based Review of Clinical and Animal Studies on Simple Obesity. Evid Based Complement Alternat Med (2019) 2019:5796381. 10.1155/2019/5796381 30854010 PMC6378065

[12] Shen W Wang Y Lu SF Hong H Fu S He S Acupuncture promotes white adipose tissue browning by inducing UCP1 expression on DIO mice. BMC Complement Altern Med (2014) 14:501. 10.1186/1472-6882-14-501 25514854 PMC4301852

[13] Lu SF Tang YX Zhang T Fu SP Hong H Cheng Y Electroacupuncture Reduces Body Weight by Regulating Fat Browning-Related Proteins of Adipose Tissue in HFD-Induced Obese Mice. Front Psychiatry (2019) 10:353. 10.3389/fpsyt.2019.00353 31244685 PMC6580183

[14] Heinonen S Jokinen R Rissanen A Pietilainen KH White adipose tissue mitochondrial metabolism in health and in obesity. Obes Rev (2020) 21(2):e12958. 10.1111/obr.12958 31777187

[15] Lee JH Park A Oh KJ Lee SC Kim WK Bae KH The Role of Adipose Tissue Mitochondria: Regulation of Mitochondrial Function for the Treatment of Metabolic Diseases. Int J Mol Sci (2019) 20(19). 10.3390/ijms20194924 PMC6801758 31590292

[16] Wu Z Puigserver P Andersson U Zhang C Adelmant G Mootha V Mechanisms controlling mitochondrial biogenesis and respiration through the thermogenic coactivator PGC-1. Cell (1999) 98(1):115–24. 10.1016/S0092-8674(00)80611-X 10412986

[17] Milne JC Lambert PD Schenk S Carney DP Smith JJ Gagne DJ Small molecule activators of SIRT1 as therapeutics for the treatment of type 2 diabetes. Nature (2007) 450(7170):712–6. 10.1038/nature06261 PMC2753457 18046409

[18] Canto C Gerhart-Hines Z Feige JN Lagouge M Noriega L Milne JC AMPK regulates energy expenditure by modulating NAD+ metabolism and SIRT1 activity. Nature (2009) 458(7241):1056–60. 10.1038/nature07813 PMC3616311 19262508

[19] Qiang L Wang L Kon N Zhao W Lee S Zhang Y Brown remodeling of white adipose tissue by SirT1-dependent deacetylation of Ppargamma. Cell (2012) 150(3):620–32. 10.1016/j.cell.2012.06.027 PMC3413172 22863012

[20] Zong H Ren JM Young LH Pypaert M Mu J Birnbaum MJ AMP kinase is required for mitochondrial biogenesis in skeletal muscle in response to chronic energy deprivation. Proc Natl Acad Sci U S A (2002) 99(25):15983–7. 10.1073/pnas.252625599 PMC138551 12444247

[21] Benador IY Veliova M Mahdaviani K Petcherski A Wikstrom JD Assali EA Mitochondria Bound to Lipid Droplets Have Unique Bioenergetics, Composition, and Dynamics that Support Lipid Droplet Expansion. Cell Metab (2018) 27(4):869–85 e6. 10.1016/j.cmet.2018.03.003 PMC5969538 29617645

[22] Fei W Tian DR Tso P Han JS Arcuate nucleus of hypothalamus is involved in mediating the satiety effect of electroacupuncture in obese rats. Peptides (2011) 32(12):2394–9. 10.1016/j.peptides.2011.10.019 22064014

[23] Tian DR Li XD Wang F Niu DB He QH Li YS Up-regulation of the expression of cocaine and amphetamine-regulated transcript peptide by electroacupuncture in the arcuate nucleus of diet-induced obese rats. Neurosci Lett (2005) 383(1-2):17–21. 10.1016/j.neulet.2005.03.039 15885905

[24] Cabyoglu MT Ergene N Tan U The treatment of obesity by acupuncture. Int J Neurosci (2006) 116(2):165–75. 10.1080/00207450500341522 16393882

[25] Keipert S Jastroch M Brite/beige fat and UCP1 - is it thermogenesis? Biochim Biophys Acta (2014) 1837(7):1075–82. 10.1016/j.bbabio.2014.02.008 24530356

[26] Enerback S Jacobsson A Simpson EM Guerra C Yamashita H Harper ME Mice lacking mitochondrial uncoupling protein are cold-sensitive but not obese. Nature (1997) 387(6628):90–4. 10.1038/387090a0 9139827

[27] Medina-Gomez G Mitochondria and endocrine function of adipose tissue. Best Pract Res Clin Endocrinol Metab (2012) 26(6):791–804. 10.1016/j.beem.2012.06.002 23168280

[28] Ohtsuji M Katsuoka F Kobayashi A Aburatani H Hayes JD Yamamoto M Nrf1 and Nrf2 play distinct roles in activation of antioxidant response element-dependent genes. J Biol Chem (2008) 283(48):33554–62. 10.1074/jbc.M804597200 PMC2662273 18826952

[29] Collu-Marchese M Shuen M Pauly M Saleem A Hood DA The regulation of mitochondrial transcription factor A (Tfam) expression during skeletal muscle cell differentiation. Biosci Rep (2015) 35(3). 10.1042/BSR20150073 PMC4613705 26182383

[30] Piantadosi CA Suliman HB Mitochondrial transcription factor A induction by redox activation of nuclear respiratory factor 1. J Biol Chem (2006) 281(1):324–33. 10.1074/jbc.M508805200 16230352

[31] Li PA Hou X Hao S Mitochondrial biogenesis in neurodegeneration. J Neurosci Res (2017) 95(10):2025–9. 10.1002/jnr.24042 28301064

[32] Vernochet C Damilano F Mourier A Bezy O Mori MA Smyth G Adipose tissue mitochondrial dysfunction triggers a lipodystrophic syndrome with insulin resistance, hepatosteatosis, and cardiovascular complications. FASEB J (2014) 28(10):4408–19. 10.1096/fj.14-253971 PMC4202105 25005176

[33] Sutherland LN Capozzi LC Turchinsky NJ Bell RC Wright DC Time course of high-fat diet-induced reductions in adipose tissue mitochondrial proteins: potential mechanisms and the relationship to glucose intolerance. Am J Physiol Endocrinol Metab (2008) 295(5):E1076–83. 10.1152/ajpendo.90408.2008 18780775

[34] Du H Zhou C Wu H Shan T Wu Z Xu B Effects of Electroacupuncture on PGC-1 alpha Expression in Brown Adipose Tissue. Evid Based Complement Alternat Med (2013) 2013:625104. 10.1155/2013/625104 24489587 PMC3892755

[35] Lu M He Y Gong M Li Q Tang Q Wang X Role of Neuro-Immune Cross-Talk in the Anti-obesity Effect of Electro-Acupuncture. Front Neurosci (2020) 14:151:151. 10.3389/fnins.2020.00151 32180699 PMC7059539

[36] Wang LH Li J Huang W Wang L Ran GP Cheng DJ [Electroacupuncture Relieves Obesity by Up-regulating PGC-1 alpha/UCP-1 Signaling in White Adipose Tissue in Diet-induced Obesity Rats]. Zhen Ci Yan Jiu (2018) 43(8):495–500. 10.13702/j.1000-0607.170744 30232852

[37] Hardie DG AMP-activated protein kinase: a cellular energy sensor with a key role in metabolic disorders and in cancer. Biochem Soc Trans (2011) 39(1):1–13. 10.1042/BST0390001 21265739

